# Global Stillbirth Policy Review – Outcomes And Implications Ahead of the 2030 Sustainable Development Goal Agenda

**DOI:** 10.34172/ijhpm.2023.7391

**Published:** 2023-08-15

**Authors:** Nana A. Mensah Abrampah, Yemisrach B. Okwaraji, Danzhen You, Lucia Hug, Salome Maswime, Caroline Pule, Hannah Blencowe, Debra Jackson

**Affiliations:** ^1^Faculty of Epidemiology and Population Health, Department of Infectious Disease Epidemiology, London School of Hygiene and Tropical Medicine, London, UK; ^2^Maternal, Adolescent, Reproductive & Child Health Centre, London School of Hygiene & Tropical Medicine, London, UK; ^3^Division of Data, Analytics, Planning and Monitoring, UNICEF, New York City, NY, USA; ^4^Global Surgery Division, Department of Surgery, University of Cape Town, Cape Town, South Africa; ^5^School of Public Health, University of the Western Cape, Cape Town, South Africa

**Keywords:** Stillbirths, Fetal Death, Perinatal Health, Measurement, Health Policies, Health Systems

## Abstract

**Background:** Globally, data on stillbirth is limited. A call to action has been issued to governments to address the data gap by strengthening national policies and strategies to drive urgent action on stillbirth reduction. This study aims to understand the policy environment for stillbirths to advance stillbirth recording and reporting in data systems.

**Methods:** A systematic three-step process (survey tool examination, identifying relevant study questions, and reviewing country responses to the survey and national documents) was taken to review country responses to the global 2018-2019 World Health Organization (WHO) Reproductive, Maternal, Neonatal, Child and Adolescent Health (RMNCAH) Policy Survey. Policy Survey responses were reviewed to identify if and how stillbirths were included in national documents. This paper uses descriptive analyses to identify and describe the relationship between multiple variables.

**Results:** Responses from 155 countries to the survey were analysed, and over 800 national policy documents submitted by countries in English reviewed. Fewer than one-fifth of countries have an established stillbirth rate (SBR) target, with higher percentages reported for under-5 (71.0%) and neonatal mortality (68.5%). Two-thirds (65.8%) of countries reported a national maternal death review panel. Less than half (43.9%) of countries have a national policy that requires stillbirths to be reviewed. Two-thirds of countries have a national policy requiring review of neonatal deaths. WHO websites and national health statistics reports are the common data sources for stillbirth estimates. Countries that are signatories to global initiatives on stillbirth reduction have established national targets. Globally, nearly all countries (94.8%) have a national policy that requires every death to be registered. However, 45.5% of reviewed national policy documents made mention of registering stillbirths. Only 5 countries had national policy documents recommending training of health workers in filling out death certificates using the International Classification of Diseases (ICD)-10 for stillbirths.

**Conclusion:** The current policy environment in countries is not supportive for identifying stillbirths and recording causes of death. This is likely to contribute to slow progress in stillbirth reduction. The paper proposes policy recommendations to make every baby count.

## Background

**Key Messages**

**Implications for policy makers**

National policy-makers should:

- Establish a standard national definition for stillbirths and include stillbirth registration as part of strategies to accelerate progress to end preventable stillbirths.
- Undertake reviews of reproductive, maternal, newborn, child and adolescent health plans and guidelines, and include specific reference to the training of health workers to record and register stillbirths and their causes according to internationally recognized standards.
- Improve the reporting infrastructure at country level with clear protocols for health workers and ensure data on stillbirths is shared between different actors and health system levels.
- Consider joining global initiatives that aim to reduce stillbirth rates (SBRs) such as the *Every Newborn Action Plan (ENAP)* and the *Quality of Care ( QoC ) Network for Maternal, Newborn and Child Health (MNCH)*.
- Ensure policies do not remain detached from frontline efforts by including adequately financed implementation plans at the facility and district levels.

The highlighted recommendations are applicable to health providers and stakeholders involved in stillbirth prevention. It is essential to ensure that policies, training and reporting infrastructure on stillbirth are available and sensitized within countries.

**Implications for the public**

Findings from 155 countries and over 800 national policy documents reveal stillbirths remain invisible in national policies. Countries that responded to the survey prioritized child health mortality indicators (such as under-five mortality rate [U5MR] and neonatal mortality rate [NMR]), three-times more than stillbirths. The regions with the highest burden of stillbirth, Africa and South-East Asia, accounted for more than half of all established stillbirth rate (SBR) targets. 40.6% of reporting countries in Africa and 21.9% of reporting countries in South-East Asia had established SBR targets. Greater than half of all reporting countries with established SBR targets are middle-income, with gaps reported in countries facing fragility, vulnerability, and conflict. Overall, more countries reported review processes for maternal (65.8%) and neonatal deaths (67.7%) compared to stillbirth (43.9%). Improving the policy environment which directs how stillbirths are acted upon at country-level is an essential step in creating the enabling environment needed to make every baby count.

Stillbirth is a global health crisis that affects millions of families each year. Globally, 1 in 72 babies are stillborn, amounting to around 2 million stillbirths annually.^1^ Over the last twenty years, the stillbirth rate (SBR) has declined by only 2.3% compared annually to a 2.9% reduction in neonatal mortality, 4.3% in mortality among children aged 1–59 months and 2.9% for maternal mortality.^1^ The stagnating trend has resulted in calls for increased investment at global and national levels. Several global publications, initiatives and networks have emerged to amplify and accelerate progress on reducing stillbirths. These include the* Every Newborn Action Plan (ENAP)*^2,3^; *Global Strategy for Women and Child Health*^4^; *the Network for Improving Quality of Care ( QoC ) for maternal, newborn, and child health (MNCH)*^5^; and the *Core Stillbirth Estimation Group of the United Nations Inter-agency Group for Child Mortality Estimation (UN IGME)*.^6^ SBR is also part of the World Health Organization (WHO) *Global Reference List of 100 Core Health Indicators. *Within ENAP, a prominent target is for countries to achieve SBRs of 12 or fewer stillbirths per 1000 total births by 2030 and to close equity gaps.

 There is an acknowledgment that the unequal gains witnessed in stillbirth compared to other MNCH outcomes require further investment.^1,2,7^ Many stillbirths are preventable through improved peri-conceptual health and nutrition, high quality antenatal and delivery care, and improved health systems.^7^ Health systems also provide the foundations needed to deliver quality care.^8^ Health systems building blocks including leadership and governance are required to drive action and investment at the point of care. Information systems allow for evidence-informed decision-making. Financing arrangements remove barriers to health service access. Essential commodities and a skilled, motivated health workforce support the delivery of QoC interventions.

 As a critical function of health systems, leadership and governance are vital roles governments play in the stewardship of health systems. The central role of governments is to provide policy guidance underpinned by oversight, collaboration and coalition, regulation, and accountability.^8^ ENAP has issued a call to action to governments to review and sharpen national strategies, policies, and guidelines for newborns and stillbirths. Prioritizing and establishing national targets for SBR reduction provides direction to sub-national and facility teams for better reporting and measurement on the neglected burden of stillbirth, drives the identification of measures to achieve the stated target, and holds governments accountable. Prioritization of stillbirths within national plans also creates awareness for health workers to document better and can drive increased investments into stillbirth measurement and reporting infrastructure.

 This paper seeks to provide an overview of the policy environment in countries to understand stillbirths recording and reporting. The policy instruments used in this paper refer to policies, strategies, laws, plans, and guidelines. Specifically, we aimed to: understand the governance related to stillbirths; assess processes established for maternal deaths, stillbirths, and neonatal deaths; identify health information systems commonly used for data collection on maternal and perinatal mortality; understand availability of essential commodities for maternal and perinatal services; explore national health workforce policies for stillbirth reporting; and finally, examine national-level policies and processes on death registration and stillbirths. The selection of objectives was informed by the WHO Health Systems Framework.^8^

## Methods

###  Design 

 The continuum of services across reproductive, maternal, neonatal, child and adolescent health (RMNCAH) is key for QoC in a country. Country responses to the global 2018-2019 WHO RMNCAH Policy Survey were reviewed to understand the policy environment for stillbirth.^13,14^ The survey, distributed to all 194 Member States of WHO via email, tracked country progress in adopting WHO recommendations in national health policies, strategies and guidelines related to RMNCAH.^13^ The survey was communicated by WHO, with an indicated timeframe, for WHO country offices to complete with relevant Ministry of Health and other United Nations (UN) agencies. Country responses to the survey were validated against national documents submitted by countries to WHO, with the required follow-up done by WHO. WHO conducted an analysis and published the results of the broader RMNCAH survey in the *International Journal of Health Policy and Management.*^15^ This report did not systematically focus on, or review critically stillbirths.

###  Survey Question Selection – Inclusion and Exclusion Process 

 The policy survey was modular and included 331 questions and associated sub-questions. Thematic areas for the survey included cross-cutting RMNCAH issues, maternal and newborn health, child health, adolescent health, reproductive health, and gender-based violence.^16^

 For inclusion in this review, the two survey modules with content on stillbirths were examined: firstly, the cross-cutting RMNCAH module, and secondly, the maternal and newborn health module.

**Figure 1:**
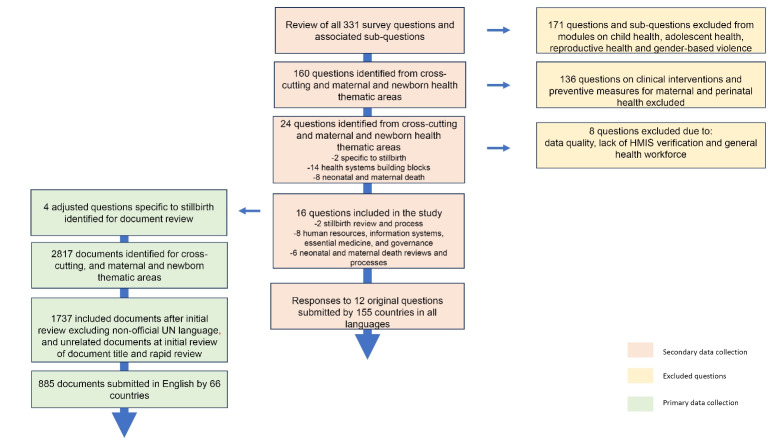
Methodology Flow Chart. Abbreviations: Abbreviations: UN, United Nations; HMIS, Health Management Information Systems.

 From the two relevant survey modules, a systematic three-step process was conducted to determine study questions to be included in the study. In the first step, we (the authors of this paper) reviewed all 160 questions and sub-questions captured within the cross-cutting, and maternal and newborn health modules of the RMNCAH policy survey questionnaire. We identified questions related to stillbirth or influencing stillbirth outcomes using three perspectives. For inclusion, first, all questions that specifically mention stillbirth. Second, questions related to health systems building blocks that are essential facilitators for creating an enabling environment for stillbirth reduction^8^; and finally, questions on stillbirth-related areas such as neonatal and maternal deaths which are highly correlated to SBR. We excluded questions about clinical interventions and preventive measures for perinatal and maternal health. Twenty-four questions were identified from this step (Figure 1).

 We submitted a data sharing request form to WHO outlining the scope and intended output of the research. We obtained from WHO, secondary data including the original country responses to the 24 questions, catalogued national policy documents submitted by countries to validate and substantiate the survey responses, protocols for validation of country survey responses against national documents, and information from WHO on any data quality concerns relating to these questions.

 As a second step, once the data was received for the 24 questions, data verification was undertaken. Three questions for which responses could not be verified through the national documents were excluded. These included a question that required verification in the national health management information system and two questions on the frequency of death review panel meetings. Five questions that addressed general human resources were dropped, as more focused responses were available in a specific question on human resources for stillbirths (See Table S1 of Supplementary file 1). National documents were reviewed to ensure that countries that indicated “yes” to established stillbirth targets had stated targets.

 Overall, 16 questions (See Table S2 of Supplementary file 1) were included in this study: one question relating to national targets for SBR, under-five mortality rate (U5MR), and neonatal mortality rate (NMR); four questions on policies for death registration processes (birth registrations were not accounted for in this study as the term is used to refer to registration of live births, not stillbirths or fetal deaths^17^); two questions on essential medicines and equipment; one question on surveys and health management information systems; and eight questions on death reviews. From the 16 questions, original country responses to 12 questions submitted by the 155 responding countries in all languages were included for the global review. For the remaining four questions, the questions had relevance to stillbirth, but stillbirth was not directly mentioned, for example, “is there a national policy/law that requires every death to be registered?” These four questions were adjusted to make them stillbirth specific eg, “is there a national policy/law that requires every death including [stillbirth or fetal death] to be registered?”

 For the third step, national documents for the sixty-six countries who submitted documents in English (Table S3, Supplementary file 1) were then examined to answer the four adjusted questions (Table S2, Supplementary file 1) using a defined search protocol. A response to the adjusted questions was then recorded. Responses to these four questions served as primary data. Search terms used for this analysis included: still, stillbirth, still birth, fetal, foetus, fetus, and foetal. Associated definitions for the search terms are reflected in Table S4 of Supplementary file 1.

 Limitations to this approach are further expanded upon in the limitations section.

###  Analysis

 The WHO Health Systems Framework was most appropriate to our study as it allows for a description of the various organizations, institutions, resources and people that work together to reduce SBRs. Past studies have also highlighted the usefulness of applying this framework to achieve health goals.^18-20^ The WHO Health Systems Framework guided the framing of study objectives and presentation of results. Country responses were recorded for each objective in a data tracking sheet to determine if stillbirth was addressed (Table S2, Supplementary file 1). These responses were then analysed using the WHO regional groupings as the primary level of analysis. Countries also identified as fragile, conflict-affected, and vulnerable (FCV) settings, and the 2021 World Bank country income classification were used as an additional level of analysis.^21,22^ STATA 16 was used for data cleaning and analyses. Descriptive analyses were used to identify and describe the results across regions.

## Results

###  A Global Perspective on National Stillbirth Policy Environment 

 The following results were obtained from 155 countries (80% of WHO Member State countries) who responded to the wider RMNCAH survey. This captured 95.2% of the current burden of stillbirths in 2019.

###  Governance for Stillbirths: Mortality Targets

**Figure 2:**
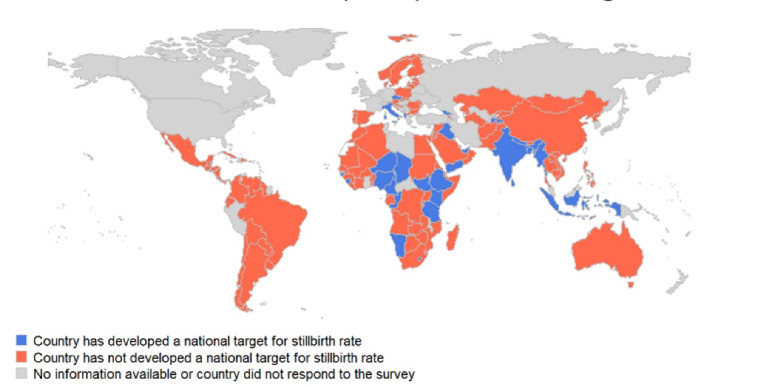
Countries That Have Developed a National Target for Stillbirth Rate, as Reported on the 2018-2019 RMNCAH Policy Survey. Abbreviation: RMNCAH, Reproductive, Maternal, Neonatal, Child and Adolescent Health.

 A national target for SBR was developed in 32 countries (21.9%). No established SBR target was reported in 114 countries and 9 countries did not respond to the question (Figure 2). Two regions accounted for over 60% of countries with a national target for SBR (Africa 40.6% [n = 13] and South-East Asia 21.9% [n = 7]). This is partly due to the large number of reporting countries in the African region.

**Figure 3:**
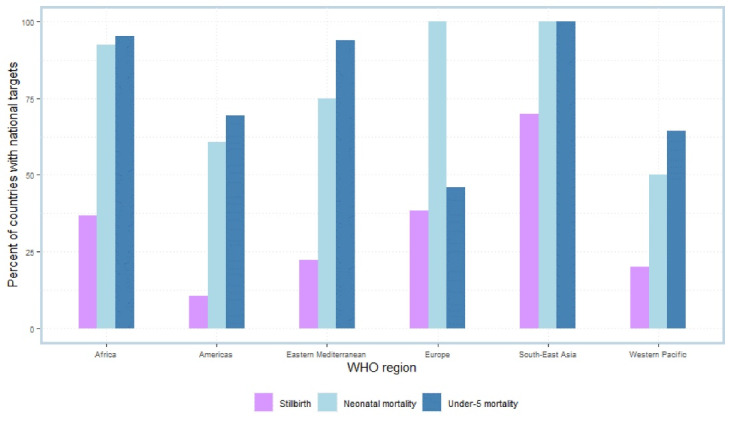
Countries That Have Developed a National Stillbirth Rate Target Compared to Neonatal Mortality Rate and Under-Five Mortality Rate Targets, Grouped by WHO Region. Abbreviation: WHO, World Health Organization.

 In comparison, three quarters of countries (n = 110) reported having set a national target for U5MR, and 68.5% (n = 102) reported a national NMR target (Figure 3). Results from the survey indicate that for countries with an established SBR target, 28.1% (n = 9) had set these greater than the ENAP target of 12 or fewer stillbirths per 1000 total births. Nearly half of all countries with identified NMR and U5MR targets, these were set at greater than the ENAP target of 12 or fewer deaths per 1000 live births and the Sustainable Development Goal target of 25 or fewer deaths per 1000 live births, respectively.

 Of the 32 countries reporting having a national target for SBR, 15.6% (n = 5) are high-income, 18.8% (n = 6) are upper-middle-income, 40.6% (n = 13) reported as lower-middle-income, and 25.0% (n = 8) are classified as low-income countries. Among the 39 globally recognized FCV countries, 29 completed the survey. A third of FCV countries who responded to the survey, reported having set a SBR target.

###  Review Processes ( eg, Panels or Committees) Established for Maternal Deaths, Neonatal Deaths, and Stillbirths

####  Review Processes Established for Maternal Deaths Including Stillbirths

**Figure 4:**
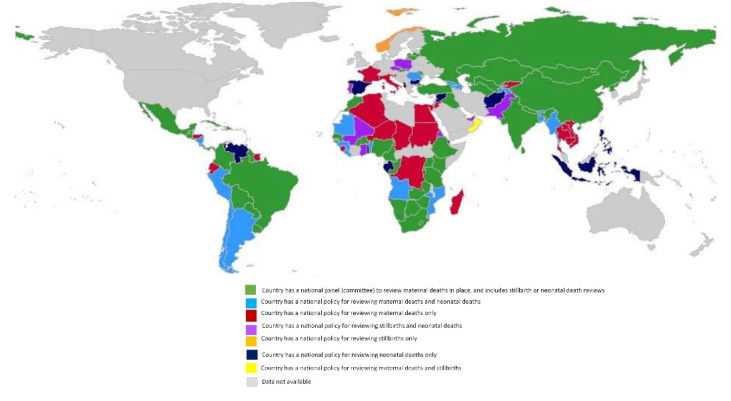
National Review Processes Established for Maternal Deaths, Stillbirths and Neonatal Deaths, as Reported on the 2018-2019 WHO RMNCAH Policy Survey. Abbreviation: RMNCAH, Reproductive, Maternal, Neonatal, Child and Adolescent Health.

 Maternal death review panels provide an opportunity to learn from the circumstances surrounding the death of a woman. Two-thirds of countries reported a national maternal death review panel or committee, and no information was available for 8.4% (n = 13) (Figure 4). Of the 102 countries with maternal death review, over half (59.8%, n = 61) reported that stillbirth and neonatal death reviews were integrated in the system. Integrated systems, defined as the investigation into a stillbirth or neonatal death should they have occurred alongside with a maternal death, were most common in Africa (39.3% of countries, n = 24), the Americas (23.0%, n = 14), Europe (13.1%, n = 8), and South-East Asia (13.1%, n = 8).

####  Review Processes Established Specifically for Stillbirths 

 A little less than half (n = 68) of countries have a national policy that requires stillbirth causes to be reviewed. Of this number, 62 countries have a policy requiring stillbirth review supported by an established operational facility-level review process. This approach was more common in Africa and the Americas compared to other regions. A further six countries reported having a national policy to review stillbirths but no facility-level review processes in place. No national policy was available in 43.9% (n = 68) of countries, however, facility-level review mechanisms exist in 13 of these countries. Only 3.9% (n = 6) of countries reported unknown or no information.

####  Review Processes Established Specifically for Neonatal Deaths 

 Two-thirds of countries (n = 105) have a national policy requiring review of neonatal deaths. Of this number, 95 countries have a national policy/guideline/law requiring neonatal death reviews alongside a facility neonatal death review process. This was common in Africa, the Americas and Europe compared to other regions. A national policy requiring neonatal death review was available in 10 countries, but no facility-level processes exist. No national policy for neonatal death review was reported in 5.2% (n = 8) of countries, however facility review processes existed. Only 3.9% (n = 6) of countries responded having no information.

####  Health Information Systems Commonly Used for Data Collection on Maternal and Perinatal Rates

**Figure 5:**
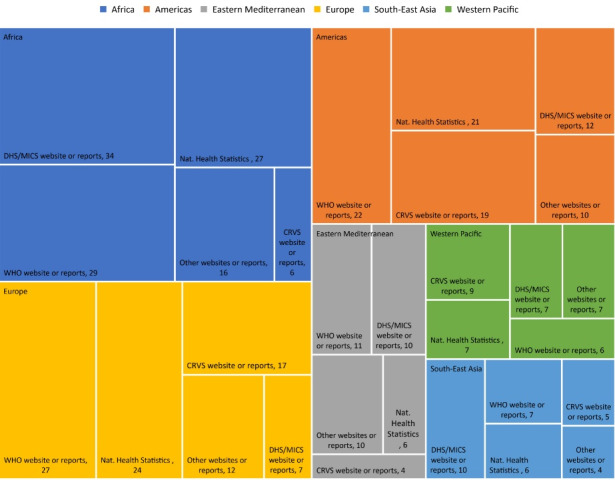
Top Five Data Sources Used by Countries to Compare Child and Maternal Mortality Rates. Each box is proportional to its value, meaning that bigger boxes have bigger proportions. Abbreviations: WHO, World Health Organization; DHS, Demographic and Health Surveys; MICS, Multiple Indicator Cluster Surveys; CRVS, Civil Registration and Vital Statistics; Nat Health statistics, National Health Statistics.

 The relevant survey question on health information systems looked into data sources for comparison. Across countries, the four most used data sources to compare maternal, newborn, child, and adolescent mortality rates, in descending order were: WHO websites and reports; national health statistics databases; national population-based surveys (eg, Demographic and Health Surveys and Multiple Indicator Cluster Surveys); and civil registration and vital statistics systems (Figure 5).

###  Essential Commodities for Quality of Care for Maternal and Perinatal Services

 Globally, more than 90.3% of countries had a national policy or guideline for essential medicines and equipment. Over 80% of countries had key commodities including oxygen supply, blood and blood products, self-inflating bag with neonatal and paediatric masks of different size, for pregnancy and childbirth care which are required for stillbirth prevention and resuscitation of babies who are apnoeic at birth and would otherwise be classified as a ‘fresh stillbirth.’

###  A Focused Perspective on National Policy Documents and Stillbirth 

 The following results are obtained from the document review of the 66 countries (out of 155) that submitted documents in English on content relevant to stillbirths, using the four stillbirth-specific adjusted questions. 885 documents (31.4% of those submitted for the cross-cutting, and maternal and newborn thematic areas) were examined as part of the primary data review.

 Among national policies reviewed, 45.5% (n = 30) mention registering stillbirths, according to established national guidelines or protocols. Just 12.1% (n = 8) of countries reported a national policy/law that requires the cause of death registration for stillbirth or fetal death to be in line with the WHO International Classification of Diseases (ICD)-10.^23^ When cause of death was mentioned for stillbirths, ICD-10 was rarely referenced. 24.3% (n = 16) of countries have a policy/law that requires a routine audit and review of death certification for stillbirth or fetal death. Across countries, when certificates were mentioned in the context of stillbirths, countries did not differentiate between death or medical certificates.

 Training of health workers in filling out death certificates using ICD-10 classification for stillbirths was reported in 5 out of the 66 countries. When training of health workers was mentioned, it generally entailed communication and counselling to the mother/family after a stillbirth.

 Between national agencies, 32% (n = 21) of countries require death data recorded on stillbirth at health facility or at the community-level be provided to the national statistics office, civil registration system, or equivalent bodies. 30.3% (n = 20) of countries required sharing individual death records on stillbirth between central and district/regional health directorate levels. Reporting stillbirth data that occurred in private facilities was mentioned by two countries in national documents.

## Discussion

 In the post Millennium Development Goals era, focus on stillbirth has improved.^24^ A coalition of agencies and initiatives including the *Network for improving QoC for MNCH, the UN IGME, ENAP, *and* the Global Strategy for Women and Child Health* are coordinating action by providing guidance on stillbirth reduction. These initiatives have exerted influence on the political priority for stillbirth.^2,3,7,25-28^ Since its launch in 2014, countries who signed onto the ENAP,^29^ committed to end preventable newborn mortality and stillbirths. ENAP is further underpinned by periodic monitoring processes to ensure countries are on track to achieve the 12 per 1000 total births goal by 2030.^30^ The success made by ENAP is clear, countries who actively report to ENAP have established stillbirth targets. Similarly, the eleven countries who are the founding members of the QoC Network for MNCH, all have established SBR targets.^5^

 In October 2020, with the launch of the global report on *A Neglected Tragedy: The global burden of stillbirths*^1^ by the UN IGME, there was consensus and acknowledgement by international agencies and networks that further work is required to include stillbirths in all relevant maternal and newborn health policies. Implementing the actions shared in the global report will require involvement of actors at the country and local levels to make sustainable improvements.

 Stillbirths are not prioritized in most countries when compared to other child health indicators.^10,12,31^ Only 21.9% of countries have established a national target for SBR (compared to 68.5% for NMR and 73.8% for U5MR), and less than half of countries have a national policy for stillbirths to be reviewed. This lower proportion of countries signals that stillbirths continue to be relegated to a “not now” agenda. Though in 2019, an estimated 2.0 million babies were stillborn roughly similar to the number of neonatal deaths.^1^ The gains reported in child health^10,12^ (2.9% reduction in neonatal mortality and 4.3% among children aged 1–59 months annually over the last 20 years) are consistent with findings from this policy review and align with the historical increase in global calls for standardized and improved measurement on newborns.^32^ An increased number of countries have established a national NMR target (and a higher number of countries reported neonatal death review processes at national and facility-level) and U5MR target.

 Some factors that play a role in why stillbirth prioritization lags at the country-level compared to other child health areas. First, definitions for stillbirths vary. Whilst standard definitions for stillbirth are included in WHO’s ICD, including a standard definition for international comparison, these definitions are not consistently applied.^33^ Universal application of these definitions is essential to enable accurate comparisons between countries and within countries over time and to identify where the need is greatest. Second, the literature on stillbirth has predominately focused on clinical interventions^34-38^ with very little information positioning the stillbirth agenda in a way that is understood by and attracts attention of policy-makers, rather than just clinicians.^7^ This affects how stillbirths are portrayed and prioritized internally within the policy community.^39^ Third, culture, taboo and misconception about stillbirths remain a big barrier.^40^

 Contextual factors play a role in how stillbirth policies and strategies are acted upon at country-level. Few (15.6%) high-income countries have established a SBR target. This may be due to increased focus being placed on low- and middle-income countries where overall national SBRs frequently remain greater than the 12 per 1000 total births ENAP targets.^1^ However, ENAP targets also include a requirement to close equity gaps in SBR in all contexts. This will require focus on the highest risk groups in every country in terms of improving equity of access and use of essential health services to end preventable deaths.^3^ In low-income countries where the risk of stillbirth is on average 7.6 times higher than in high-income countries,^1^ 25% of low-income countries in this study have set a SBR target. This is likely due in part to more active engagement in ENAP and active WHO and the United Nations International Children’s Emergency Fund (UNICEF) support of country implementation and monitoring of ENAP in high mortality settings, including support for target setting.^3,27,28^ For low-income countries that have not set a stillbirth target, these countries could benefit from increased investments into stillbirth policy setting and data strengthening. Further, national policies and strategies to reduce stillbirths would benefit from scaling up QoC interventions, which are often the same interventions to reduce maternal and neonatal mortality and improve well-being.^1,7^

 In FCV settings where health systems face complexity with service delivery resourcing, organization, access, and use,^22,41,42^ 29 countries responded to the survey. The 10 countries classified as FCV settings with an established SBR target are all signatories to the ENAP, signaling commitment by national governments to prevent newborn deaths and stillbirths. Some FCV settings do not have an established stillbirth target. This can be attributed to the significant health and development needs, competing priorities, service delivery disruption, and unpredictable resourcing streams witnessed in such settings.^41-43^ To achieve the global target of 12 or less stillbirths per 1000 total births by 2030, careful attention will need to be given to FCV settings, which account for significant health burden globally.^43^ This will require accelerating access to essential quality care^44^; improving health workforce competencies^45^; addressing systems redesign, infrastructure and essential commodities in health facilities and at the community level^41-43^; and improving data on stillbirths to address inequities in these settings.

 National policies need to be grounded by operational mechanisms at the sub-national and facility-level to facilitate the achievement of national goals.^46,47^ Setting a national approach to review stillbirths is a step in the right direction by countries, with 40% of all countries indicating that they have national policies to review stillbirth grounded by facility-level review processes to understand the causes and address gaps in service delivery, to improve QoC. A small number of countries (n = 13) indicated that there are facility-level review processes but no national policies. Contributing factors to the differences reported within countries could include varying forms of decentralized health planning and management systems within countries, which may result in greater delegation of power, community participation, and flexibility in planning to address urgent health needs in a local community^47,48^; extended time to translate policies into practice^49^; sub-national commitment and ownership to curb SBRs; and funding from global-level initiatives and point-of-care efforts to improve SBRs.^50^

 Globally, 56 countries are off track to meet the SBR ENAP target by 2030.^51^ At the country-level, several challenges remain to record and report a stillbirth: First, due to the varied definitions on stillbirths, countries use different criteria, including gestational age and birthweight for stillbirth measurement.^37,52^ Second, misclassification of antepartum and intrapartum stillbirths,^53,54^ differentiating between intentional late term induced abortion and stillbirth,^55^ and distinguishing stillbirth and early neonatal death,^11,56-58^ further compromises data quality of stillbirth reporting. Third, challenges persist in reporting stillbirths within health information systems. Only 32% of surveyed countries require death data recorded on stillbirths at health facilities or by community health workers be provided to the national statistics office, civil registration system, or equivalent bodies. This finding warrants further research at country-level to understand and close the evidence-gap on how the policy environment affects reporting of stillbirths into health information systems. When data is reported on stillbirth, noted challenges include under-reporting, omission or misclassification of deaths in civil registration systems,^59^ and limited information on birthweight, gestational age, and stillbirth type in the health management information systems.^60,61^ Further, socio-cultural and spiritual beliefs in some countries are identified barriers for mothers underreporting stillbirths or hindering families to register a stillbirth in demographic health surveys.^40,62^

 A key intervention proposed to improve stillbirth recording and reporting are death reviews. Maternal and perinatal death surveillance and response (MPDSR) has expanded in recent years,^63-66^ with a view to learning about causes and promoting successful partnerships at different levels that can lead to real change for communities and nations. Globally, only 39.4% of countries have identified the inclusion of stillbirth or neonatal death reviews as part of national committees on maternal death reviews, resulting in missed opportunities for an efficient and integrated review process alongside identifying the high-yield QoC intervention packages to save both mother and baby. Further missed opportunities were reported where very few countries had mechanisms to review causes of death for stillbirth. Some reasons for countries reporting no review or lack of an integrated review process may include policy and planning environment, resource support, historical focus by external actors on the implementation of MPDSR, political prioritization, pressures to implement, and the level of connectedness and networks between health system levels.^64,66^

 Stillbirths remain invisible in most national documents. Less than half of reviewed national policy documents made mention of registering stillbirths and just 12.1% made mention of identifying stillbirth causes according to ICD-10. A systematic classification^67,68^ such as ICD-10, supports national tracking, provides in-depth investigation, grounds research, and identifies areas of greatest need. Low utilization of classification systems in facilities may be due to scarce national resourcing thereby affecting coverage and lack of required data.^68^ Several studies have called on training of health workers on management related to stillbirth^40,69,70^ to address the gaps related to health worker skills for perinatal death reviews. Properly identifying the causes of stillbirth is important for women to know why their baby died, to reduce blame and stigma and take positive action for the next pregnancy.^71^

###  Limitations 

 This study has some limitations. The study is a policy review and does not assess the level of implementation of the various policies. Additionally, the document reviews were conducted only in English. Documents submitted in the other five official UN languages were excluded, in addition to those in local national languages. Though the document reviews, were only conducted in English, this was the highest share of documents submitted in official UN languages. We recommend that further reviews in other UN official languages be conducted to augment these findings.

 Reliability of country responses can be a problem as this is based on the knowledge of the individuals reporting the data at country-level. The situation in the country may have changed since the time of the survey with new guidelines having been released from WHO on MPDSR (2021) and the COVID-19 pandemic. The nature in which questions were framed within the WHO RMNCAH survey may have influenced the responses by country. For example, “what is the target” does not indicate if it is a current or future target.

###  Recommendations

 The following recommended actions for policy-makers could improve prioritization of stillbirths within national policies and plans, ahead of the 2030 Sustainable Development Goal deadline. First, close the large gaps in stillbirth registration by using a standard definition for stillbirths and explicitly incorporating stillbirth into RMNCAH policies and plans. The WHO ICD-11 (released 2022) is now updated with a revised standard definition for stillbirth.^72^ Where stillbirth is not included, include stillbirth registration as part of plans for stillbirth reduction. Second, undertake reviews of RMNCAH plans and guidelines, with a specific reference to the training of health workers to record and register stillbirths and their causes according to internationally recognized standards such as ICD-11. This action should apply to all health facilities including public and private facilities and at the community-level. Third, develop simple communication and advocacy materials making the case for stillbirth policy improvements for policy-makers. Fourth, improve the reporting infrastructure at the country level with clear protocols for health workers and ensure data on stillbirths is shared between different actors. Data is needed to develop sound policies. Finally, ensure policies do not remain detached from frontline efforts by including adequately financed implementation plans at the facility and district levels.

 At the global level, we suggest improvements to the WHO RMNCAH policy survey to address the urgent need for stillbirth reduction and better reporting, including a dedicated thematic area on stillbirth. Integration of the additional four adjusted questions on stillbirth into the WHO RMNCAH policy survey could provide a better picture of the policy landscape for stillbirths and allow for useful information for policy tracking in addition to the data collected by the ENAP. We suggest that international agencies increase investments in stillbirth by expanding upon countries participating in global initiatives (such as ENAP and the QoC Network) to advance the stillbirth agenda. We also urge global and implementing partners to provide guidance and training for how governments can incorporate stillbirths in national policies and plans on RMNCAH and strengthen data systems to record and report on stillbirths.

## Conclusion

 Networks and global initiatives play a key role in supporting the policy environment to reduce stillbirths. The findings from this global policy review highlight great gaps exist in setting national direction for stillbirth reduction. Without improving the policy environment which directs how stillbirths are acted upon at country-level, the global goal of reducing the SBR to 12 or less stillbirths in every country per 1000 total births will remain aspirational.

## Acknowledgements

 We gratefully acknowledge the contributions of Allyson Moran and Elizabeth Katwan. Further thanks to the Stillbirths Advocacy and Research in Africa Hub (SARAH). We also acknowledge the country survey respondents and WHO for enumeration of the survey, cleaning of data and cataloguing the national source documents that enabled this review. No specific funding was provided for the analyses in this paper, however, funding for DJ was provided by the Takeda Foundation as part of the Takeda Chair in Global Child Health at the London School of Hygiene and Tropical Medicine.

## Ethical issues

 Ethics approval was received from the London School of Hygiene and Tropical Medicine to conduct the policy review. Additionally, we submitted a data sharing request form to WHO outlining the scope and intended output of the research. Approval was obtained and we received from WHO all national policies and documents submitted to the global 2018-2019 WHO RMNCAH Policy Survey.

## Competing interests

 Authors declare that they have no competing interests.

## Supplementary files

Supplementary file 1 contains Tables S1-S4.
