# Supplementary material for: Global Stillbirth Policy Review – Outcomes And Implications Ahead of the 2030 Sustainable Development Goal Agenda
Source: Int J Health Policy Manag. 2023 Aug 15;12:7391. doi: 10.34172/ijhpm.2023.7391 (PMC10590256; doi:10.34172/ijhpm.2023.7391)
Supplement: Supplementary file 1 — contains Tables S1-S4. [file ijhpm-12-7391-s001.pdf]

Article title: Global Stillbirth Policy Review – Outcomes And Implications Ahead of the 2030 Sustainable Development Goal Agenda

Journal name: International Journal of Health Policy and Management (IJHPM)

Authors' information: Nana A. Mensah Abrampah[1]*, Yemisrach B. Okwaraji[2], Danzhen You[3], Lucia Hug[3], Salome Maswime[4], Caroline Pule[4], Hannah Blencowe[2], Debra Jackson[2,5]

***Correspondence to**: Nana A. Mensah Abrampah, Email: nana.mensah-abrampah@lshtm.ac.uk

Citation: Mensah Abrampah NA, Okwaraji YB, You D, et al. Global stillbirth policy review – outcomes and implications ahead of the 2030 Sustainable Development Goal agenda. Int J Health Policy Manag. 2023;12:7391. doi:10.34172/ijhpm.2023.7391

Supplementary file 1

Table S1. Rationale for excluding eight (out of the 24 questions) questions

	ID	Thematic Area	RMNCAH Policy Question	Rationale for excluding
1	MN_23	Childbirth Policy	Does the country have a national policy/guideline on the right of every woman to have access to skilled care at childbirth?	Focused question on human resources for stillbirths
2	CC_57	Surveys and health management information systems	Does your national health information system (HIS) collect and report on the following data?	Requires verification in national health management information systems
	CC_57e		Number or rates of live births	
	CC_57f		Number or rates of stillbirths	
	CC_57g		Number or rates of newborn deaths?	
	CC_57h		Causes of newborn death?	
3	MN_68	Human Resources Policy	Are there national policies/guidelines that set forth a competency framework for maternal and/or newborn health care?	Focused question on human resources for stillbirths

4	MN_69	Human Resources Policy	Is there a continuous professional education system in place for primary health-care clinicians and/or nurses to receive maternal and/or newborn-specific training?	Focused question on human resources for stillbirths
5	MN_70	Human Resources Policy	Is there a national policy/guideline on education of midwifery care providers based on International Confederation of Midwives (ICM) competencies?	Focused question on human resources for stillbirths
6	MN_71	Human Resources Policy	Is there a national policy/guideline on regulation of midwifery care providers (doctors, nurses, and midwives) based on ICM competencies?	Focused question on human resources for stillbirths
7	MN_88	Maternal deaths	How often does the national panel (committee) meet?	Frequency of death review panel meetings
			Monthly	
			Quarterly	
			Semi-annually	
			Annually	
			Unknown	
8	MN_89	Maternal deaths	When did the panel last meet?	

Table S2. Guidance to reviewing national documents, original and adjusted questions

- **Step 1**: Open the main folder with all available documents.
- **Step 2**: Review the excel sheet named *Metadata_WHO_SRMNCAH_PolicySurvey_2018_2019_data.xlsx* with the original survey responses provided by countries to the selected questions. This is the secondary data information collected by WHO.
- **Step 3:** Open the primary data collection sheet with the adjusted questions (see below table) and familiarize yourself. This sheet is the one needing completion.
- **Step 4**: Open the link for the national documents to be reviewed. The national documents are grouped by WHO regional office categorization and then country listing.
- **Step 5:** For the adjusted questions in the primary data collection sheet, for each country, review all the national documents that are in English with the associated search words: still, stillbirth, still birth, fetal, foetus, fetus, foetal. The review of national documents will be conducted using the associated search terms. For example, once a national document is open, use the command: ctrl + f (for windows) or command + f (for Mac) to search the document. Further definitions are provided in table S4.
- **Step 6**: Record the response for the primary question, following review of national level documents. Use the guidance provided before recording the response.
    - If stillbirth is identified as addressed per question in the national document, include 1 for yes in the excel sheet labelled *adjusted_primary_data*
    - If stillbirth is not specifically mentioned in national documents, enter 0 for no in the *adjusted_primary_data* sheet.
    - If no national documents is available, place 99 in the cell.
    - If the national document is not in English, place 2 in the cell.
- **Step 7:** Under the folder "Documents relating to stillbirth specifically" add any national policy/plan/guideline/strategy that stillbirth is mentioned. Note: the folder is grouped by region.

	ID	Thematic Area	RMNCAH Policy Survey Original Question	This study adjusted question	Associated study objective
**Module 1: Cross-cutting**					
1	CC_11b	Introductory	Has your country developed a national target for any of the following indicators?<br>[Under-five mortality rate]	Not applicable	Objective 1
			Target [per 1000 live births]		
	CC_11c	Introductory	Has your country developed a national target for any of the following indicators?<br>[Neonatal mortality rate]	Not applicable	Objective 1
			Target [per 1000 live births]		
	CC_11d	Introductory	Has your country developed a national target for any of the following indicators?<br><br>[Stillbirth rate]	Not applicable	Objective 1
			Target [per 1000 live births]		
2	CC_51	Policies on death registration processes	Is there a national policy/law that requires every death to be registered?	Is there a national policy/law that requires every death including [stillbirth or fetal death] to be registered?	Objective 6
3	CC_52	Policies on death registration processes	Does the policy/law require cause of death registration to be in line with ICD-10?	Does the policy/law require cause of death registration [for stillbirth or fetal death] to be in line with ICD-10?	Objective 6
4	CC_53	Policies on death registration processes	Is there a policy/law that requires routine audit and/or review of death certification for maternal, perinatal, neonatal and/or child deaths?	Is there a policy/law that requires routine audit and/or review of death certification for [stillbirth or fetal death]?	Objective 6
5	CC_54	Policies on death registration processes	Does the policy/law that requires routine audit and/or review of death certification do any of the following?	Not applicable	Objective 4<br>Objective 6

	CC_54a		Require the issuance of medical certificates of cause of death?	Require the issuance of medial certificates of cause of death for [stillbirth or fetal death]?	
	CC_54b		Recommend training health workers in filling out death certificates using the International Classification of Diseases (ICD)?	Recommend training health workers in filling out death certificate using the ICD-classification for stillbirth?	
	CC_54c		Require death data recorded at health facilities or by community health workers (CHWs) to be provided to the national statistics office, civil registration system, or equivalent bodies?	Require death data recorded on [stillbirth or fetal death] at health facilities or by community health workers to be provided to the national statistics office, civil registration system or equivalent bodies	
	CC_54d		Require sharing individual death records within the health system and between central and district/regional levels?	Require sharing individual death record on [stillbirth or fetal death] within the health system and between central and district/regional levels?	
	CC_54e		Recommend verbal autopsy on community deaths for determining cause of death?	Recommend or use verbal autopsy on [stillbirths or fetal death] at the community level for determining cause of deaths?	
6	CC_59	Surveys and health management information systems	What are the three most commonly used data sources to compare maternal, newborn, child, and adolescent mortality rates in your country to mortality rates in other countries?<br><br>National Health Statistics<br>Civil Registration and Vital Statistics<br>Population-based survey<br>WHO website or reports<br>UNICEF website or reports	Not applicable	Objective 3

			UN SDG website or reports<br>WB website or reports<br>UNDP website or reports<br>UNFPA website or reports<br>Institute for Health Metrics Global Burden of Disease<br>Countdown to 2030 website or reports<br>Other		
Module 3: Maternal and Newborn Health					
7	MN_75	Essential Medicines and Equipment	Are there national policies/guidelines on essential medicines and equipment?	Not applicable	Objective 4
8	MN_77	Essential Medicines and Equipment	Are any of the following supplies and equipment included in the national list of commodities indicated for use of pregnancy, childbirth and postpartum care?<br><br>Obstetric ultrasound machine?<br>Self-inflating bag (newborn size) with neonatal and paediatric masks of different size and valve?<br>Oxygen supply?<br>Pulse oximeter?<br>Blood and blood products?<br>Vacuum aspiration?	Not applicable	Objective 4
9	MN_84	Maternal deaths	Is there a national panel (committee) to review maternal deaths in place?	Not applicable	Objective 2
10	MN_87	Maternal deaths	Does this national panel (committee) include stillbirth or neonatal death reviews? [Y/N]	Not applicable	Objective 2
11	MN_90	Maternal deaths	Is/are there a subnational panel(s) (committee(s)) to review maternal deaths in place?	Not applicable	Objective 2

12	MN_92	Stillbirths	Is there a national policy/guideline/law that requires stillbirths (fresh or macerated) to be reviewed?	Not applicable	Objective 2
13	MN_93	Stillbirths	Is there a facility stillbirth review process in place?	Not applicable	Objective 2
14	MN_95	Neonatal death	Is there a national policy/guideline/law that requires neonatal deaths (0-28 days) to be reviewed?	Not applicable	Objective 2
15	MN_96	Neonatal death	Is there a national policy requiring classification of the causes of stillbirths and neonatal deaths according to the ICD-PM (WHO application of ICD-10 to deaths during the perinatal period) classification?	Not applicable	Objective 2
16	MN 97	Neonatal death	Is there a facility neonatal death review process in place?	Not applicable	Objective 2

Table S3 – 885 documents submitted in English by 66 countries for review

WHO Region	Arabic	Chinese	English	French	Not Official Language	Russian	Spanish	Grand Total
Europe			98	30	689	50	15	882
Americas			77	14	52		464	607
Africa			211	205	47			463
Eastern Mediterranean	91		175	32				298
Western Pacific		24	174		99			297
South-East Asia			150		120			270
Grand Total	91	24	885	281	1007	50	479	2817

Table S4. ***Operational definitions***

Classification	Description
Fetal death	*Fetal death is intrauterine death prior to the complete expulsion or extraction from a woman of a fetus irrespective of the duration of pregnancy.*
Stillbirth	*Stillbirth is the complete expulsion or extraction from a woman of a fetus following a fetal death at 22 or more completed weeks of gestation; or if gestational age is not available with a birthweight of 500 grams or more.*
Antepartum stillbirth	*Complete expulsion or extraction from a woman of a fetus following an antepartum fetal death at 22 or more completed weeks of gestation; or if gestational age is not available with a birthweight of 500 grams or more.*
Intrapartum stillbirth	*Complete expulsion or extraction from a woman of a fetus following an intrapartum fetal death at 22 or more completed weeks of gestation; or if gestational age is not available with a birthweight of 500 grams or more.*
Fresh stillbirth	*Complete expulsion or extraction from a woman of a fetus following a fetal death at 22 or more completed weeks of gestation; or if gestational age is not available with a birthweight of 500g or more with skin showing no signs of maceration (fresh appearance).*
Macerated stillbirth	*Complete expulsion or extraction from a woman of a fetus following a fetal death at 22 or more completed weeks of gestation; or if gestational age is not available with a birthweight of 500g or more with skin showing signs of maceration.*<br>*Maceration describes the degenerative changes that occur in stillbirths retained in the utero after death, and the earliest signs are in the form of discoloration and peeling of the skin, leaving regions of raw tissue* [[1]].

[1] World Health Organization, *ICD-11 for Mortality and Morbidity Statistics* 2020: Geneva
